# Timely diagnosis and staging of non‐alcoholic fatty liver disease using transient elastography and clinical parameters

**DOI:** 10.1002/jgh3.12385

**Published:** 2020-06-29

**Authors:** Christine Shieh, Dina L Halegoua‐De Marzio, Matthew L Hung, Jonathan M Fenkel, Steven K Herrine

**Affiliations:** ^1^ Division of Gastroenterology and Hepatology Thomas Jefferson University Hospital Philadelphia Pennsylvania USA; ^2^ Department of Radiology Hospital of the University of Pennsylvania Philadelphia Pennsylvania USA

**Keywords:** fibrosis, non‐alcoholic fatty liver disease, screening, transient elastography

## Abstract

**Background and Aim:**

There is no standardized guideline to screen, image, or refer patients with non‐alcoholic fatty liver disease (NAFLD) to a specialist. In this study, we used transient elastography (TE) to examine the fibrosis stages at which patients are first diagnosed with NAFLD. Subsequently, we analyzed metabolic markers to establish cut‐offs beyond which noninvasive imaging should be considered to confirm NAFLD/non‐alcoholic steatohepatitis fibrosis in patients.

**Methods:**

Charts spanning July 2015–April 2018 for 116 NAFLD patients who had TE performed were reviewed. Univariate and multivariate analysis of metabolic markers was conducted.

**Results:**

At the first hepatology visit, TE showed 73% F0–F2 and 27% F3–F4. Univariate analysis showed that high‐density lipoproteins (HDL), hemoglobin A1c (A1c), aspartate transaminase (AST), and alanine transaminase (ALT) were significantly different between the F0–F2 and F3–F4 groups. Multivariate analysis showed that AST (*P* = 0.01) and A1c (*P* = 0.05) were significantly different. Optimal cut‐offs for these markers to detect liver fibrosis on TE were AST >43 U/L and A1c >6.6%. The logistic regression function combining these two variables to reflect the probability (*P*) of the patient having advanced fibrosis (F3–F4) on TE yielded the formula: *P* = *e* ^*R*^/(1 + *e* ^*R*^), where *R* = −8.56 + 0.052 * AST + 0.89 * A1c.

**Conclusions:**

Our study suggested that >25% of patients presenting to a specialist for NAFLD may have advanced fibrosis (F3–F4). Diabetes (A1c >6.6%) and AST >43 U/L were the most predictive in identifying NAFLD patients with advanced fibrosis on imaging. We proposed a formula that may be used to prioritize NAFLD patients at higher risk of having advanced fibrosis for specialist referral and imaging follow‐up.

## Graphical abstract

In this study, NAFLD patients at a tertiary care center are stratified by fibrosis stage and a logistic regression analysis is conducted to identify common metabolic markers most predictive of high risk NAFLD patients. We establish laboratory value cutoffs for the two most significant biomarkers to help guide timely and appropriate referral of NAFLD/NASH patients from primary care setting to a specialist.

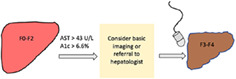

## Introduction

Non‐alcoholic fatty liver disease (NAFLD) and its more severe inflammatory form, non‐alcoholic steatohepatitis (NASH), are two of the most common etiologies of liver disease in the United States today. Around 10% of patients with NAFLD will progress to NASH, with up to 25% of this subset developing cirrhosis and half of the subset dying from associated complications.^1^ In recent years, cross‐sectional imaging, such as ultrasound (US) and magnetic resonance (MR) elastography, has emerged as a safe and reproducible noninvasive way to evaluate the severity of NAFLD/NASH by using low‐frequency vibrations to measure stiffness of the organ in units of kilopascals (kPa) and translating these units into different degrees of fibrosis.^2^ First validated to detect cirrhosis in viral hepatitis, US elastography (USE) is now used to detect fibrosis in other chronic liver conditions.^3^, ^4^ Several studies have established elastography as an accurate means to evaluate liver fibrosis in patients with NASH.^5^ A meta‐analyses of 50 studies assessed the performance of USE for diagnosis of liver fibrosis and found an increased predictive value of kPa with increase in fibrosis severity grouped as F0–F2 and F3–F4 across different etiologies of chronic liver disease.^6^ Elastography was the best at differentiating F0–F3 from F4, but a combination of USE with biomarkers has been theorized to improve diagnostic accuracy.^6^

NAFLD/NASH is often asymptomatic, so initial suspicion for the condition can follow incidental findings of abnormal liver enzyme levels.^7^ Of patients, 80% may have normal transaminase levels or elevated transaminase levels that fall back to normal as fibrosis progresses to cirrhosis.^8^ Given the subtle ways in which patients can present with NAFLD/NASH, there is concern that this condition is being underdiagnosed.^9^ A recent study of a large European database suggested that the general population was being diagnosed at later stages of the disease.^10^

The aim of our retrospective study was twofold. First, given the nonstandardized manner in which NAFLD/NASH is diagnosed in the primary care setting, we conducted a cross‐sectional analysis to investigate which stage of liver fibrosis patients appear to have on USE by the time they present to a specialist for suspected NAFLD/NASH. Second, we performed a univariate and multivariate analysis on serum metabolic markers of this patient population to establish lab value cut‐offs beyond which noninvasive imaging such as USE should be considered by future providers to more promptly evaluate patients for significant NAFLD/NASH fibrosis.

## Methods

### *Sample selection*

This is a retrospective cross‐sectional study on 116 adult patients aged 23–79 years who were referred to a hepatologist at a tertiary care center for NAFLD/NASH between July 2015 and April 2018. In order to focus solely on patients with NAFLD/NASH without other confounding etiologies of liver disease, patients who had chronic liver disease from an etiology other than NAFLD/NASH, such as alcohol (defined as >7 drinks/week for females and >14 drinks/week for males), viral hepatitis, and biliary disease, were excluded. All charts of included patients were reviewed to verify that the diagnosis of NAFLD/NASH was correctly assigned and were not fibrosis of other etiologies misclassified as NASH. The study was approved by the institutional review board (IRB) at our center. All patients underwent US‐based transient elastography (TE) using FibroScan. The following demographic, clinical, and laboratory data were recorded: age, gender, BMI, glucose, albumin, lipid panel, hepatic function panel, platelets, and hemoglobin A1c (A1c).

### *Transient elastography*

All patients had TE performed by an experienced operator. An exam was considered reliable if interquartile range (IQR) was less than 15%. A patient was considered to have F0–1 fibrosis if kPa was 2.5–7, F2 if kPa was 7.1–9.5, F3 fibrosis if kPa was 9.6–12.5, and F4 if kPa was >12.5.^9^ These cut‐offs have been validated across prior studies that examined the use of elastography to detect different stages of liver fibrosis.^6^, ^11^ Thirty‐three patients also had a liver biopsy performed as part of their workup. The pathologist at our institution graded fibrosis based on the NAFLD Activity Score (NAS).^10^, ^12^

### *Statistical analysis*

Statistical analysis was performed using GraphPad and R. Significance level was set at *P* = 0.05. Univariate analysis among categorical variables was performed using the unpaired *T*‐test. Variables that were significant on univariate analysis were included in multivariate analysis. Area under the receiver operating characteristic curve (AUROC) was used to validate a variable's ability to distinguish between degrees of fibrosis. Youden index was used to estimate appropriate cut‐off values for significant variables.^13^ Logistic regression was used to estimate a probability equation reflecting the combination of significant variables on multivariate analysis.

## Results

### *Patient characteristics*

**Figure 1:**
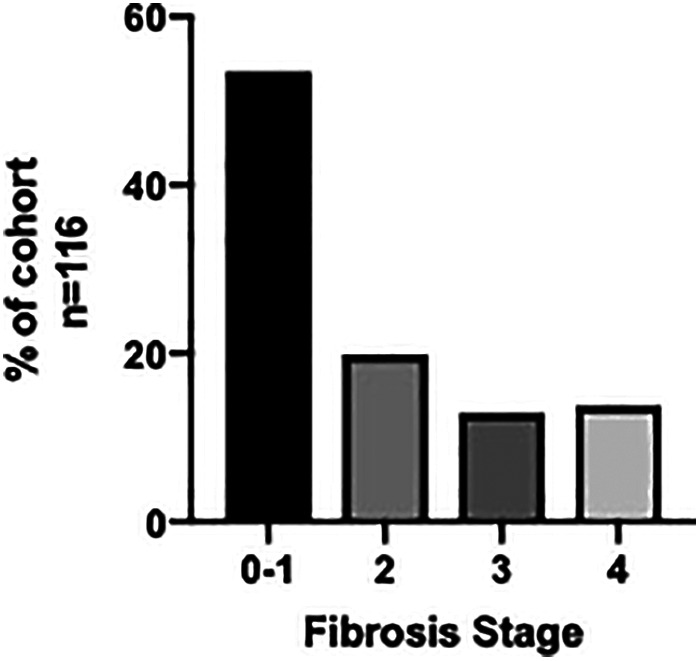
Transient elastography results (in kPa) at initial hepatology appointment (*n* = 116) showed 53% F0–F1, 20% F2, 13% F3, and 14% F4.

A total of 116 patients were included in the study. Mean age was 52.6 ± 14.4 years, with 64.6% female and 35.4% male. TE was performed using FibroScan and, at the first hepatology appointment, showed that 73% had stages F0–F2 fibrosis (*n* = 85), while 27% had stages F3–F4 fibrosis (*n* = 31) **(**Fig. 1 **)**. Ten of the patients in the F0–F2 group and two of the patients in the F3–F4 group had some alcohol use recorded. Although not every patient had a liver biopsy, elastography fibrosis staging did correlate with histological results in 76.0% of the 33 patients who underwent a liver biopsy with documented results **(**Table 1 **).** Correlation was defined as being classified within the same F0–F2 or F3–F4 category when comparing elastography results to biopsy results.

**Table 1:** Correlation between transient elastography (TE) fibrosis staging and liver biopsy fibrosis staging (*n* = 33); 25 of 33 (76%) correlated, while 8 of 33 (24%) did not

Patient with liver biopsy	TE fibrosis stage	Biopsy fibrosis stage	Correlation within F0–F2 and F3–F4 grouping (yes/no; more/less severe compared to TE)
1	2	1a	Yes
2	2	1a	Yes
3	2	2	Yes
4	0–1	1a	Yes
5	0–1	0	Yes
6	0–1	0	Yes
7	0–1	1b	Yes
8	2	2	Yes
9	0–1	1c	Yes
10	0–1	3	No; more severe
11	0–1	2	Yes
12	0–1	2	Yes
13	2	1	Yes
14	2	4	No; more severe
15	2	0	Yes
16	0–1	2	Yes
17	0–1	1	Yes
18	2	1a	Yes
19	0–1	1b	Yes
20	0–1	1a	Yes
21	4	4	Yes
22	3	2	No; less severe
23	3	2–3	Yes
24	4	4	Yes
25	4	4	Yes
26	4	2	No; less severe
27	3	2	No; less severe
28	3–4	2–3	Yes
29	4	4	Yes
30	3	1b	No; less severe
31	4	2	No; less severe
32	4	1b	No; less severe
33	4	4	Yes

For the majority of those that did not correlate, the liver biopsy results showed less severe fibrosis than TE results.

*Significance value of *p* < 0.05.

### *Univariate and multivariate analysis between* *F0–F2 and* *F3–F4 groups*

Objective parameters, including BMI, lipid levels, liver enzyme levels, platelet, albumin, and A1c, were recorded for each patient. Univariate analysis between F0–F2 and F3–F4 group showed significant differences in aspartate transaminase (AST) (*P* < 0.0001), alanine transaminase (ALT) (*P* = 0.0009), high‐density lipoproteins (HDL) (*P* = 0.0304), and A1c (*P* < 0.0001) levels. Multivariate analysis excluding patients with missing data showed significant differences in AST (*P* = 0.01; OR = 1.13) and A1c (*P* = 0.05; OR = 2.18) levels. There was no difference in BMI, low‐density lipoproteins (LDL), alkaline phosphatase (ALK), platelets (PLT), and albumin levels (Table 2).

**Table 2:** Univariate and multivariate analysis of laboratory parameters between F0–F2 and F3–F4 groups

Parameter	F0–F2 *n* = 85	F3–F4 *n* = 31	Univariate analysis *P*‐value	Multivariable analysis *P*‐value
Body mass index	31.32 ± 6.10	34.00 ± 6.93	0.054	
AST	32.44 ± 14.9	59.45 ± 38.3	<0.0001*	0.01*
ALT	45.09 ± 27.8	69.97 ± 45.5	0.0009*	0.08
ALK	76.06 ± 24.9	78.21 ± 25.3	0.70	
HDL	51.21 ± 16.9	42.77 ± 8.22	0.0304*	0.42
LDL	107.5 ± 31.1	111.1 ± 35.9	0.66	
A1c	6.07 ± 0.73	7.17 ± 1.88	<0.0001*	0.05*
PLT	234 ± 61.2	213 ± 68.2	0.16	
Albumin	4.4 ± 0.1	4.4 ± 0.33	0.84	

Fibrosis staging was evaluated through transient elastography.

Alkaline phosphatase (ALK), alanine transaminase (ALT), aspartate transaminase (AST), high‐density lipoproteins (HDL), hemoglobin A1c (A1c), low‐density lipoproteins (LDL), platelets (PLT).

*Significance value of *p* < 0.05.

### *Predictive value of* *AST* *and A1c*

**Figure 2:**
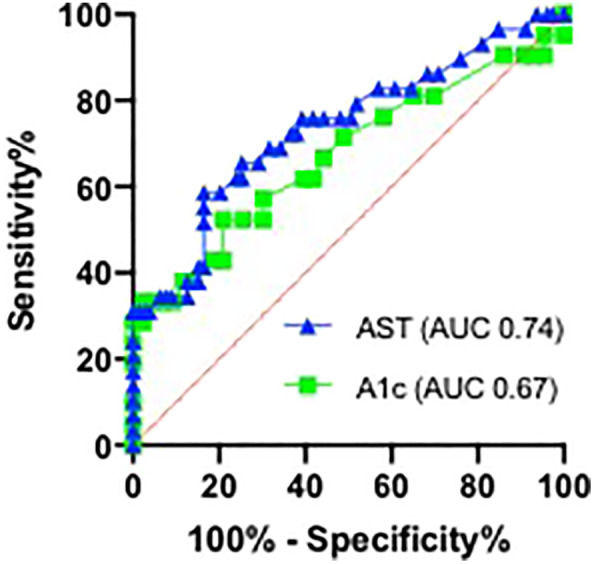
Displayed are the receiver operating characteristic curves for AST and A1c for detection of F0–F2 *versus* F3–F4 stages of fibrosis on transient elastography. 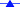, AST (AUC 0.74); 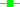, A1c (AUC 0.67).

The AUROC curve was used to evaluate the ability of AST and A1c to discriminate between F0–F2 and F3–F4 patients (Fig. 2). The area under the curve (AUC) was significant at 0.74 for AST and 0.67 for A1c. The Youden index established optimal cut‐offs at AST >43 U/L (84% specificity, 59% sensitivity) and A1c >6.6% (79% specificity, 52% sensitivity) as being predictive for having F3–F4 fibrosis on TE. At our institution, the upper limit of normal for AST is 42, and the standard cut‐off for diagnosis of diabetes by A1c is an A1c >6.5%. Of the 38 patients in our cohort who were confirmed Type 2 diabetics, 17 were found to have F3–F4 on TE. Logistic regression analysis was used to estimate an equation combining the two significant variables, AST and A1c, where P equals the probability of advanced fibrosis F3–F4 on TE and *e* is Euler's number 2.72.

$$P=e^{R}/\left(1+e^{R}\right),\text{where}\;R=-8.56+0.052*\mathrm{AST}+0.89*A1c$$

## Discussion

NAFLD is a growing epidemic in the United States. Analysis of liver ultrasound data between 1988 and 1994 reported that 19% of adults had NAFLD, while a meta‐analysis of studies from 2006 to 2014 estimated an increased NAFLD prevalence of 24%.^14^ Among NASH cases in 2015, an estimated 20% had F3/F4.^14^ This significant percentage of advanced fibrosis in NAFLD patients is corroborated by our study results, which showed that 27% of NAFLD patients may already be at advanced stages of fibrosis (F3–F4) based on USE by the time they see a hepatologist. This high prevalence of potential fibrotically advanced cases reflects untimely diagnosis of NASH from primary care practices and delayed referral to a specialist.^15^ NAFLD with advanced fibrosis has been established as an independent predictor of mortality risk.^16^ This population of patients is therefore important to identify early on in the course of the disease.

In order to more promptly identify NAFLD patients with potentially significant fibrosis from the primary care setting, laboratory markers must be used to help guide decision‐making on when to order noninvasive imaging and refer to a specialist for follow‐up. Various scores and algorithms have been developed that include a wide variety of markers from basic liver function panels to proteins such as γ‐GT or α2‐macroglobulin, indicative of liver fibrosis.^17^ Our study examined the most common labs that would be screened for in a primary care setting and found that AST and A1c are the most predictive in distinguishing which patients are most likely to have advanced fibrosis (F3–F4) on noninvasive imaging and therefore should be considered for further workup and close follow‐up. Specifically in our cohort, the Youden index elicited an AST in the upper limits of normal and A1c beyond the diabetes cut‐off of 6.5% as the most specific and indicative markers of this at‐risk population. Errors in our study include small sample size after excluding for multifactorial causes of cirrhosis and the lack of confirmatory liver biopsy for every single patient, given the risks involved with a biopsy and the validated use of TE in assessing fibrosis stages across all chronic liver diseases.^6^ Nonetheless, our results reflect the recent emphasis of how the NAFLD epidemic is growing in parallel with that of Type 2 diabetes. Prevalence of advanced NASH fibrosis in diabetics has been commented on in various studies, with Bazick *et al*. recording a prevalence as high as 41.0%.^18^ Lai *et al*. explored the prevalence of NAFLD and advanced fibrosis among T2DM patients and found 72% to have NAFLD and 21% to have advanced fibrosis.^19^ Comparatively, in our cohort, 44% of the Type 2 diabetic patients were found to have F3–F4 fibrosis on TE, thereby reinforcing the idea that diabetics are at higher risk for progression of NAFLD.^20^

To help providers more promptly identify NAFLD patients at risk of advanced fibrosis in clinical settings such as endocrine or primary care clinics, we established an equation to calculate the probability that a NAFLD patient will have stages F3–F4 fibrosis on noninvasive imaging based on their AST and A1c. While other scores and algorithms have been published, each has its own pros and cons. The NAFLD Liver Fat Score and the BARD score do not distinguish between stages of fibrosis.^17^ Scores such as the BAAT score and the FIB‐4 score do not include a diabetic parameter, despite diabetes becoming significantly associated with NAFLD.^17^ The FibroTest involves a multitude of biomarkers that realistically are not screened for in a primary care setting.^17^ In fact, a cross‐sectional survey of primary care clinicians reflected very limited awareness of various scores such as the NAFLD Fibrosis Score, FIB‐4 Score, APRI Score, and the ELF test.^21^ Future work will involve validating our equation in a larger cohort and determining which probability cut‐off is the most specific for significant fibrosis in a NAFLD patient.

In conclusion, the main goal of our study was to identify laboratory parameters most indicative of advanced fibrosis on noninvasive imaging in patients suspected to have NAFLD. To that end, AST >43 and A1c >6.6% appear to be most indicative in our cohort. These parameters should be routinely screened for in primary care or algorithmically incorporated into the electronic medical record through our proposed algorithm to help provide better guidance on which patients should be evaluated by TE for fibrosis secondary to NAFLD. In this way, NAFLD patients most at risk of having significant liver fibrosis can be more promptly identified and be referred to an appropriate specialist for follow‐up.

## Declaration of Conflict of Interest

Dina L. Halegoua‐De Marzio is a consultant for Gilead and Interept. She also receives research grant support from BMS, Intercept, Genfit, Gilead, Inventiva, and Galmed. Jonathan M. Fenkel is a consultant for Gilead. He receives research grant support from Abbvie, Conatus, Gilead, and HCV‐Target. None of these companies or organizations had a role in the study design, data collection, analysis, or publication of this manuscript.

## Author contributions

Christine Shieh and Dina L. Halegoua‐De Marzio designed the study. Christine Shieh and Matthew L. Hung analyzed and interpreted the data. Christine Shieh drafted the article. All were involved in critical revision of the article.
